# Role of Platelet Parameters on Sudden Sensorineural Hearing Loss: A Case-Control Study in Iran

**DOI:** 10.1371/journal.pone.0148149

**Published:** 2016-02-01

**Authors:** Abbas Mirvakili, Mohammad Hossein Dadgarnia, Mohammad Hossein Baradaranfar, Saeid Atighechi, Vahid Zand, Abdollah Ansari

**Affiliations:** Otorhinolaryngology Research Center, Department of Otolaryngology, Head & Neck Surgery, Shahid Sadoughi University Of Medical Sciences, Yazd, Iran; School of Medicine, Fu Jen Catholic University, TAIWAN

## Abstract

Sudden sensorineural hearing loss (SSNHL) is a common otological disorder characterized by a hearing loss greater than 30 dB over three consecutive frequencies, in less than 72 hours. It has been established that platelet parameters, such as mean platelet volume, are associated with ischemic heart events, whose clinical manifestations are similar to those of SSNHL. Hence, we aimed to determine if the platelet count, mean platelet volume and platelet distribution width are related to the occurrence and severity of sudden sensorineural hearing loss. A case-control prospective study was conducted in a teaching hospital in Iran. One hundred-eight patients with SSNHL and an equal number of healthy, age- and sex-matched controls were enrolled in the study. Peripheral venous blood samples were collected from the subjects, and the platelet count, mean platelet volume and platelet distribution width were measured with an automated blood cell counter. Analysis of the audiometry and hematological test results using SPSS_22_ software showed no statistical correlation between the platelet parameters and the occurrence of SSNHL, but correlation coefficients showed a significant correlation between PDW and hearing loss severity in patients group. However, further investigation is required to unequivocally establish the absence of correlation between the platelet parameters and occurrence of SSNHL.

## Introduction

Hearing loss is a common, escalating disorder worldwide, which has multiple negative consequences on the afflicted. Studies have shown that about 50% of people are affected by hearing loss, which increases with age. The occurrence of hearing loss has seen an increase in the past decades, especially in developing countries, with its incidence doubling between 1965 and 1994 [1–3].

Sudden sensorineural hearing loss (SSNHL) is a subset of sudden hearing loss that has a sensory-neural origin [4]. SSNHL, identified by Dekleyn in 1944, is a common otological emergency, defined as sudden hearing loss of more than 30 db in at least three consecutive frequencies in a standard pure tone audiogram (PTA). SSNHL usually reaches its maximum peak rapidly. Patients suffering from this disorder often experience a sudden hearing loss at first, quickly progressing to sudden drops in hearing [1,5–7]. Only 10–15% of the SSNHL cases have specified causes, while more than 85% are of unknown etiology—idiopathic sudden sensorineural hearing loss (ISSNHL) [3,8]. The severity of hearing loss in SSNHL can vary from a minor disturbance in hearing to complete deafness, categorized clinically as low, medium or high [8].

Several reports have detailed the prevalence of SSNHL in various countries [5,8–12]. Recent epidemiological studies show an increase in the incidence of this condition [13]. It has been suggested that the exact prevalence of this condition is much higher than reported due to its high rate of spontaneous recovery without treatment [14,15].

Sudden sensorineural hearing loss occurs at all ages, with childhood incidences being rare. The lowest incidence in adults has been reported to be among the age group of 20–30, and the highest among 50–60 [1,8]. The prevalence of SSNHL is not significantly different between men and women [8]. Although the condition can occur in all seasons, conflicting reports about the seasonality of the incidence of SSNHL have been published [16,17].

Sudden sensorineural hearing loss presents symptoms such as tinnitus, dizziness with 10% of these patients experiencing dizziness with nausea and vomiting, fullness of the ear, a sense of ear congestion, vestibular disorders, feeling of pressure in the ears, headaches, and symptoms of a viral infection of the upper respiratory tract. Patients also might complain of anxiousness, stress and depression [4,8,11,18,19].

Many aspects of SSNHL are still unknown, and its etiology, risk factors, prognostic factors and treatment protocols remain controversial [5,20]. Probable etiologies of SSNHL have been suggested by various authors including infection, cardiovascular causes, immunological disorders, damage to the tympanum, genetic disorders and mutation, inheritance factors, systemic stress, autoimmune disorders, side effects from ototoxic medications such as salicylates and aminoglycosides, damage to the ears due to aging, perinatal complications, tumors of the inner ear, exposure to loud noise, temporal arthritis, coagulation disorders, local histamine production, neoplasm, prothrombotic risk factors, and the unusual effects of some surgical procedures such as cardiopulmonary bypass surgery [1,5,6,8–10,14,21–25]. However, each of these etiologies have either been confirmed or rejected in follow-up studies [26]. So far, some studies have been conducted on the correlation between various platelet parameters (including platelets count, mean platelet volume and platelet distribution width) and the occurrence of SSNHL such as Seo et al. (2014), Blaha et al (2014), Ozcan et al (2014), Ulu et al (2013), Segit et al (2013) and Karli et al. (2013) [5,14,27–30], but the results have been inconsistent.

Mean platelet volume (MPV) is a potential indicator of the production rate, size, activity, and function of platelets. Larger platelets are more active in terms of both metabolic and enzymatic activity, and more likely to aggregate than the smaller platelets [5,14,31]. Studies have shown a correlation between MPV and some clinical events such as cardiovascular ischemic events, heart attacks, stroke, vascular thrombosis, autoimmune diseases, and inflammatory conditions [5,14]. Platelets count and platelet distribution width (PDW) are other important platelet parameters [32,33]. PDW reflects the variation in size of platelets in a blood sample [34].

The aim of this study was to investigate the relationship of platelet parameters (platelet count, mean platelet volume and platelet distribution width) with the occurrence and severity of sudden sensorineural hearing loss. Therefore in this study we chose to analyze the following variables:

1. Sudden sensorineural hearing loss (SSNHL): We chose this variable for our study because although SSNHL is a common otological disorder with an increasing rate of incidence worldwide but to date its many aspects are still unknown and need to be studied more.
2. Platelets parameters including PC, MPV and PDW: We chose to analyze the role of platelets parameters on SSNHL because some authors have claimed that the occurrence and severity of SSNHL could be correlated with platelets parameters due to its similar clinical manifestation to that of vascular diseases. However, so far this likely correlation has been investigated in few studies to our knowledge. So, we think that it is a research area where we need more information.

## Materials and Methods

A prospective case-control study was conducted in the Shahid Sadoughi hospital, Yazd, Iran, between 2013 and 2015. One hundred-eight patients with SSNHL and the same number of healthy age- and sex-matched subjects were enrolled as subjects for the study. The study population consisted of patients referred to the ENT department of hospital. An informed written consent in the native language (Persian) was obtained from all the participants in the study, and the researchers were committed to maintaining confidentiality of participant information. Approval for this study was obtained from the Medicine School of Shahid Sadoughi University of Medical Sciences, Yazd, Iran, the ENT department of Shahid Sadoughi hospital, Yazd, Iran and the ethical committee of Shahid Sadoughi University of Medical Sciences, Yazd, Iran.

Patients with inflammation, infection, renal failure, chronic liver disease, pulmonary thromboembolism, chronic obstructive pulmonary disease (COPD), systemic hypertension, cardiovascular disease, diabetes mellitus, metabolic syndrome, hyperlipidemia, statin usage, obstructive sleep apnea, rheumatological diseases, inflammatory bowel disease, hematological or endocrine diseases, history of smoking, otological disease or surgery or trauma to the ear or head, were excluded from the study.

An audiometry evaluation was performed for the chosen test subjects. All evaluations were performed by the same hearing therapist, and with the same audiometer. Audiometry evaluations included pure-tone audiometry (PTA), speech audiometry (including SDS and SRT), and Immittance criteria (including tympanometry and stapedius reflex).

Pure-tone audiometry is the standard and most common type of hearing test which measures the air and bone conduction thresholds for each ear in a set of standard frequencies from 250hz to 8000hz. The test is conducted in a sound booth using a pair of headphones connected to an external audiometer. The result of the test is an audiogram diagram which plots a person's hearing sensitivity at the tested frequencies. In this study, first, each patient was instructed about the procedure. Then the earphone was placed on the ear and checked for secure and proper fit. Then the pure tones at different frequencies were presented to the patient from the low to high intensity and the lowest audible intensity was defined as the patient’s threshold for that particular frequency which is marked as such on the audiogram. This procedure was repeated for all test frequencies (250, 500, 1000, 2000, 4000 and 8000 hz) in one ear and then again in the other ear. This procedure established an air conduction pure tone threshold curve for each ear. Then the bone conduction audiometry was done. For this, the probe was placed on the mastoid, the same procedure as the air conduction audiometry was done and the bone conduction pure tone threshold curve was established for each ear.

SRT (speech reception threshold) which also called speech recognition threshold is the minimum intensity in decibels at which a patient can understand 50% of spoken words and can repeat them correctly. In this test, a number of spondaic words are presented to the patient at different intensities. Spondaic words, or spondees, are words containing two syllables that are equally accented or emphasized when they are spoken to the patient. In this study, we presented 4 spondaic words to each patient at different intensities and the lowest intensity in which the patient repeated 50% of presented spondees correctly was defined as SRT. The SRT was recorded in db for each ear, separately.

Speech discrimination testing, also known as “word understanding” or “word recognition” is another important audiological test. The aim of this test is to assess how well an individual can understand words which are fully audible based on the level of hearing (as measured via SRT). In this study, 25 unfamiliar one-syllable words were presented to each patient at 40 db above SRT. The percentage of words which patient understood and repeated correctly was recorded as Speech Discrimination Score (SDS) for each ear separately.

Tympanometry is an examination used to test the condition of the middle ear and mobility of the eardrum (tympanic membrane) and the conduction bones by creating variations of air pressure in the ear canal. It is an objective test of middle-ear function. It is not a hearing test, but rather a measure of energy transmission through the middle ear. Tympanometry is a valuable component of the audiometric evaluation. In evaluating hearing loss, Tympanometry permits a distinction between sensorineural and conductive hearing loss, when evaluation is not apparent via Weber and Rinne testing. In this test, a tone of 226 Hz is generated by the Tympanometer into the ear canal, where the sound strikes the tympanic membrane, causing vibration of the middle ear, which in turn results in the conscious perception of hearing. Some of this sound is reflected back and picked up by the instrument. Most middle ear problems result in stiffening of the middle ear, which causes more of the sound to be reflected back. Admittance is how energy is transmitted through the middle ear. The instrument measures the reflected sound and expresses it as admittance or compliance, plotting the results on a chart known as a Tympanogram. Normally, the air pressure in the ear canal is the same as ambient pressure. Also, under normal conditions, the air pressure in the middle ear is approximately the same as ambient pressure since the eustachian tube opens periodically to ventilate the middle ear and to equalize pressure. In a healthy individual, the maximum sound is transmitted through the middle ear when the ambient air pressure in the ear canal is equal to the pressure in the middle ear. In our study, after an examination of the ear with an otoscope to ensure that the path to the eardrum is clear and that there is no perforation, the test was performed by inserting the Tympanometry probe in the ear canal of patient. The instrument changed the pressure in the ear, generated a pure tone, measured the eardrum responses to the sound at different pressures and produced a Tympanogram.

The acoustic reflex (also known as the stapedius reflex, middle-ear-muscles (MEM) reflex, attenuation reflex, or auditory reflex) is an involuntary muscle contraction that occurs in the middle ear in response to high-intensity sound stimuli or when the person starts to vocalize. This reflex is a normal defensive mechanism for inner ear against the loud sounds. The acoustic reflex threshold (ART) is the sound pressure level from which a sound stimulus with a given frequency will trigger the acoustic reflex which can be measured in each ear with Tympanometry. In our study, the stapedius reflex was tested with Tympanometry for each ear of each patient and the acoustic reflex threshold was recorded.

In this study, after the performing of audiometry evaluation, the subjects who had developed SSNHL within 72 hours were entered into the study as the patient group. All the patients had a minimum 30 dB hearing loss at three consecutive frequencies. For analyzing the severity of hearing loss according to platelet parameters, the patient group was divided into 4 subgroups based on the severity of hearing loss as mild (<40 db), moderate (40–55 db), sever (55–90 db) and profound (>90 db) hearing loss. The control group participants were selected from the non-otologic patients of the ward. The exclusion criteria for the control subjects were similar to those of patient group. Therefore, patients with inflammation, infection, renal failure, chronic liver disease, pulmonary thromboembolism, chronic obstructive pulmonary disease (COPD), systemic hypertension, cardiovascular disease, diabetes mellitus, metabolic syndrome, hyperlipidemia, statin usage, obstructive sleep apnea, rheumatological diseases, inflammatory bowel disease, hematological or endocrine diseases, history of smoking or surgery or trauma to the ear or head were excluded from the control group.

Venous blood samples were collected from all the participants in tubes containing Ethylenediaminetetraacetic acid (EDTA) at 7:30 am, following an overnight fast. Mean platelet volume (MPV), platelet distribution width (PDW) and platelet count (PC) were measured for each of the blood samples. All measurements were performed with an automated blood cell counter, within 15–30 minutes after sampling, in order to avoid platelet swelling. All measurements were carried out in duplicates, and the mean values were analyzed. Data analysis was conducted using the SPSS_22_ (SPSS Inc., Chicago, IL) software. The data were summarized using descriptive statistics. Box and scatter plots were used to present descriptive statistics. An independent sample T-test, ANOVA, Pearson correlation coefficient and regression analysis were used to analyze the data. A two-tail value of p<0.05 was considered statistically significant.

## Results

The male:female ratios of the two groups were 61:47 (Table 1). The difference in the mean age of participants of the two groups was not significant. Participants with SSNHL were 45.15±14.42 years of age, and those of the control group, 43.15±11.54 years. The body mass indices were similar for both the groups. All demographic characteristics of study participants are presented in Table 1.

**Table 1:** Demographic characteristics of study participants.

Variable	Patients group (n = 108)	Control group (n = 108)	p-value
Age (years)	45.15 ± 14.42	43.15 ± 11.54	0.262
Gender (n):			
Male	61 (56.5%)	61 (56.5%)	1.000
Female	47 (43.5%)	47 (43.5%)	
BMI*	23.49±1.67	23.22±1.63	0.234

*Body Mass Index

The platelet parameters that were tested showed no significant variation between the patient and control groups. The clinical laboratory results of patients and control groups including the values of PC, MPV and PDW are presented in Table 2:

**Table 2:** Clinical characteristics of study participants.

Variable	Patients group (n = 108)	Control group (n = 108)	p-value
PC (1000/uL)	228.51±62.45	222.86±36.80	0.418
MPV (fL)	10.02±0.76	9.85±0.67	0.088
PDW (fL)	12.45±1.50	12.11±1.24	0.076

As shown in Table 2, none of the three examined parameters including PC, MPV and PDW had a significant relationship with the occurrence of SSNHL. However, we performed a logistic regression to solidify our conclusions about the relationship of platelet parameters and the occurrence of SSNHL. The results of this analysis are presented in Table 3.

**Table 3:** Regression analysis of the relationship between platelet parameters and occurrence of SSNHL

	B	Sig.
**PC (1000/uL)**	0.003	0.258
**MPV (fL)**	0.208	0.467
**PDW (fL)**	0.114	0.446

**Fig 1:**
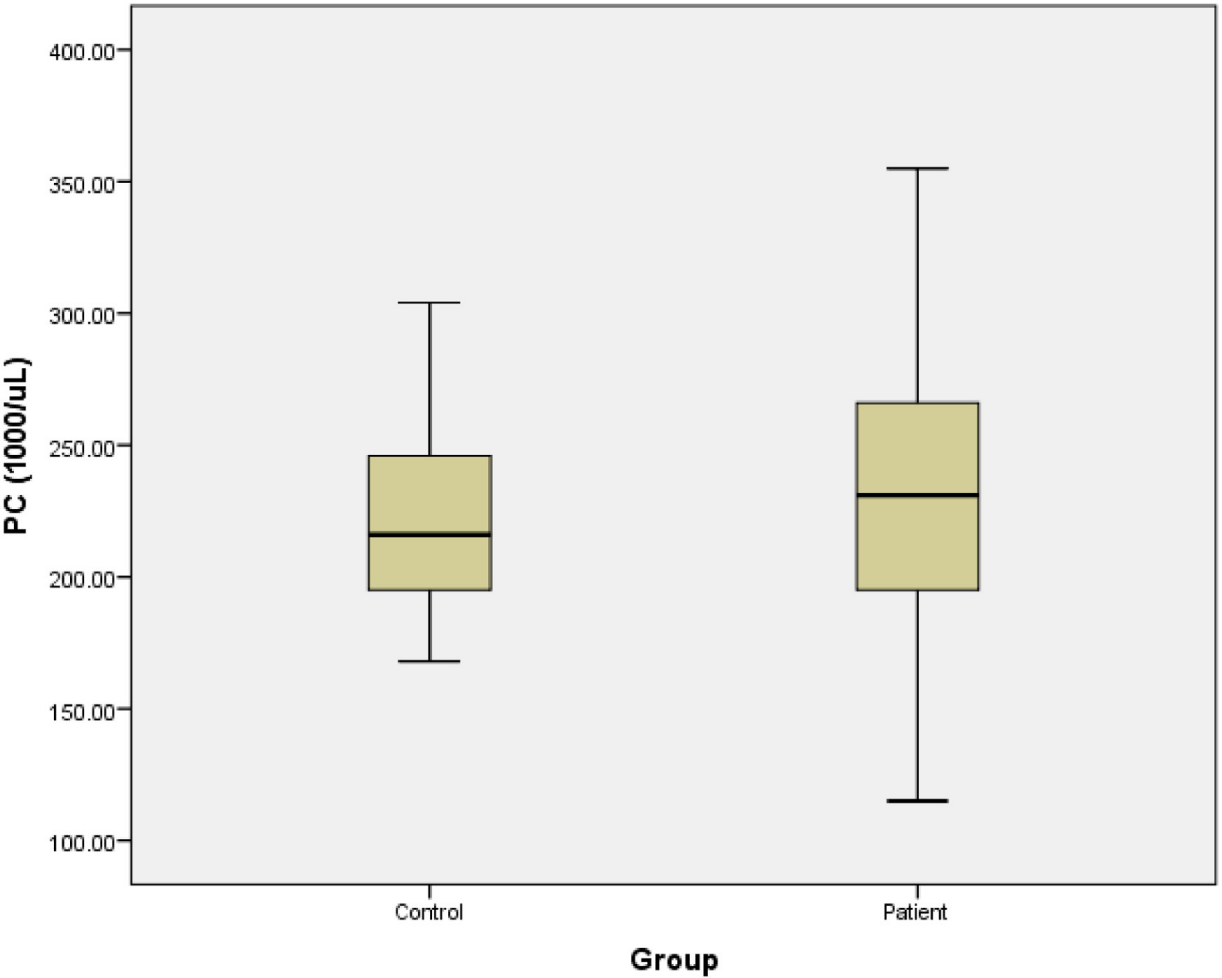
Platelets counts of patients and control groups.

**Fig 2:**
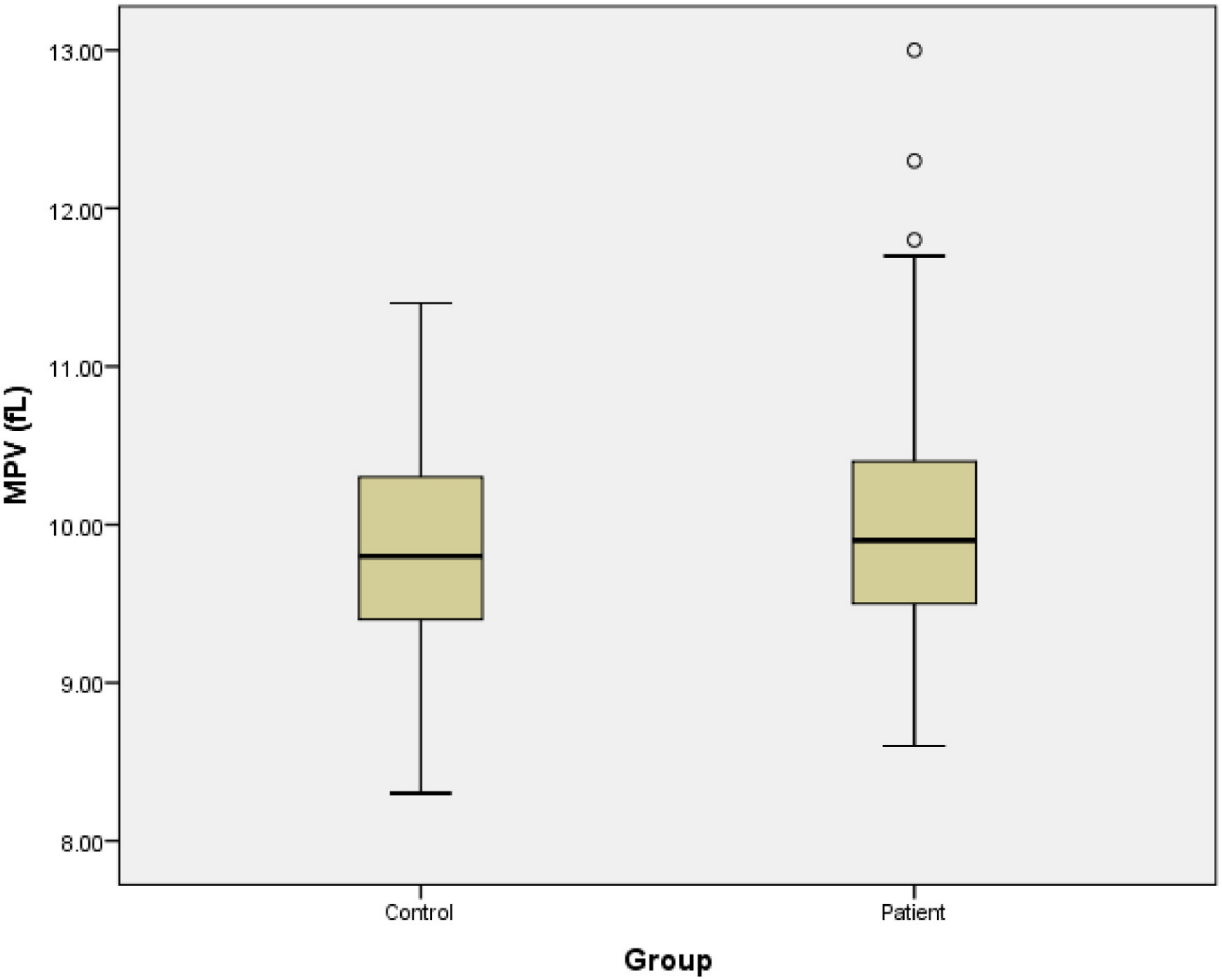
Mean platelet volumes of patients and control groups.

**Fig 3:**
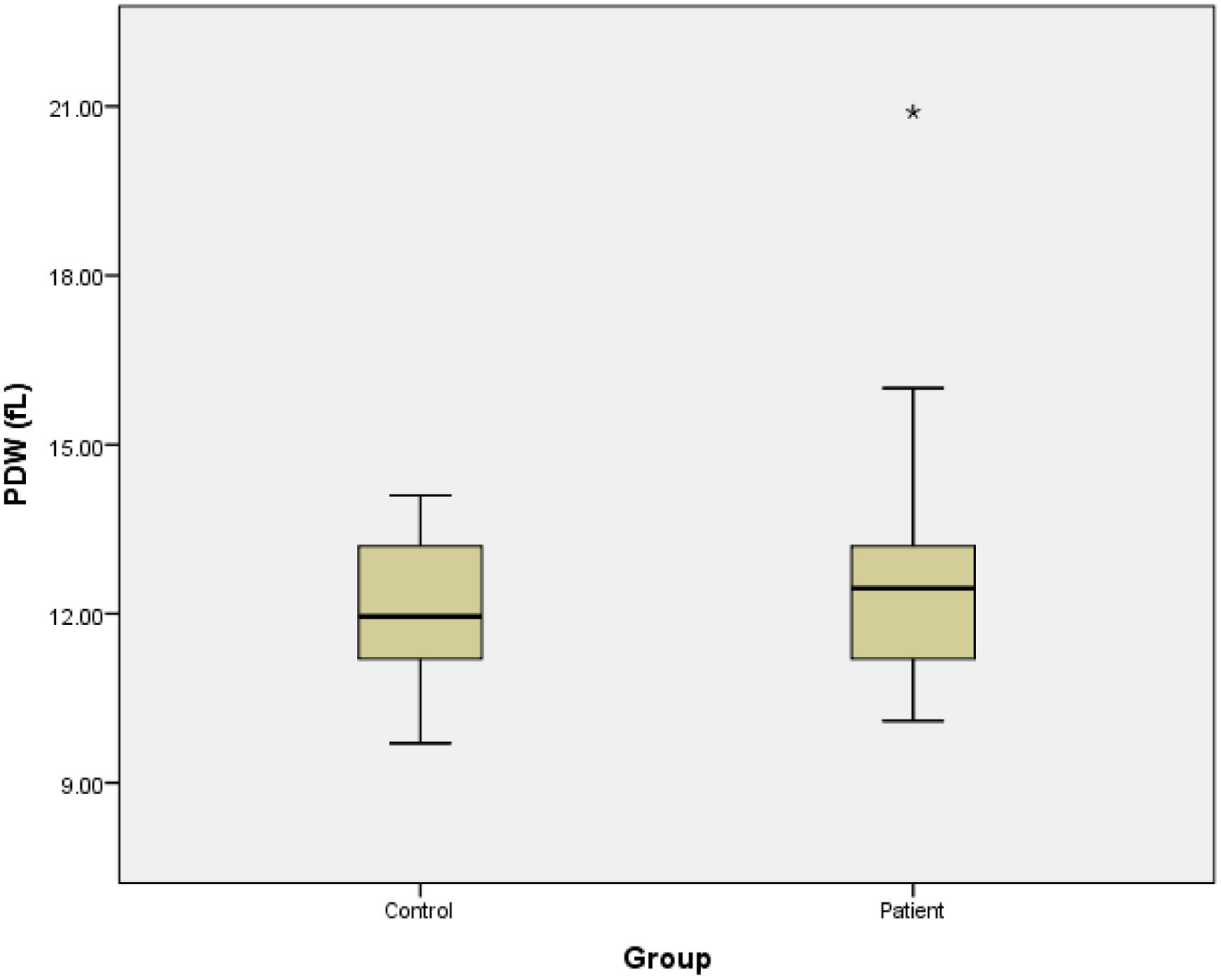
Platelet distribution widths of patients and control groups.

As presented in Table 3, the regression analysis confirmed the absence of significant relationship between platelet parameters and the occurrence of SSNHL. Also, we draw the boxplot of platelet parameters for patients and control groups. Fig 1 shows the platelet count of 2 groups. Figs 2 and 3, represent the MPV and PDW of 2 groups, respectively.

Also, the analysis of platelet parameters according to severity of hearing loss is presented in Table 4:

**Table 4:** Comparison of platelet parameters according to severity of hearing loss as mild, moderate, sever and profound.

Hearing loss Platelet parameters	Mild (n = 13)	Moderate (n = 37)	Sever (n = 51)	Profound (n = 7)	p-value
PC (1000/uL)	214.23±59.08	229.59±61.81	229.90±61.45	239.28±87.04	0.824
MPV (fL)	9.86±0.66	9.90±0.72	10.14±0.82	10.05±0.71	0.416
PDW (fL)	11.58±1.35	12.23±1.12	12.76±1.72	12.95±1.15	0.043

**Fig 4:**
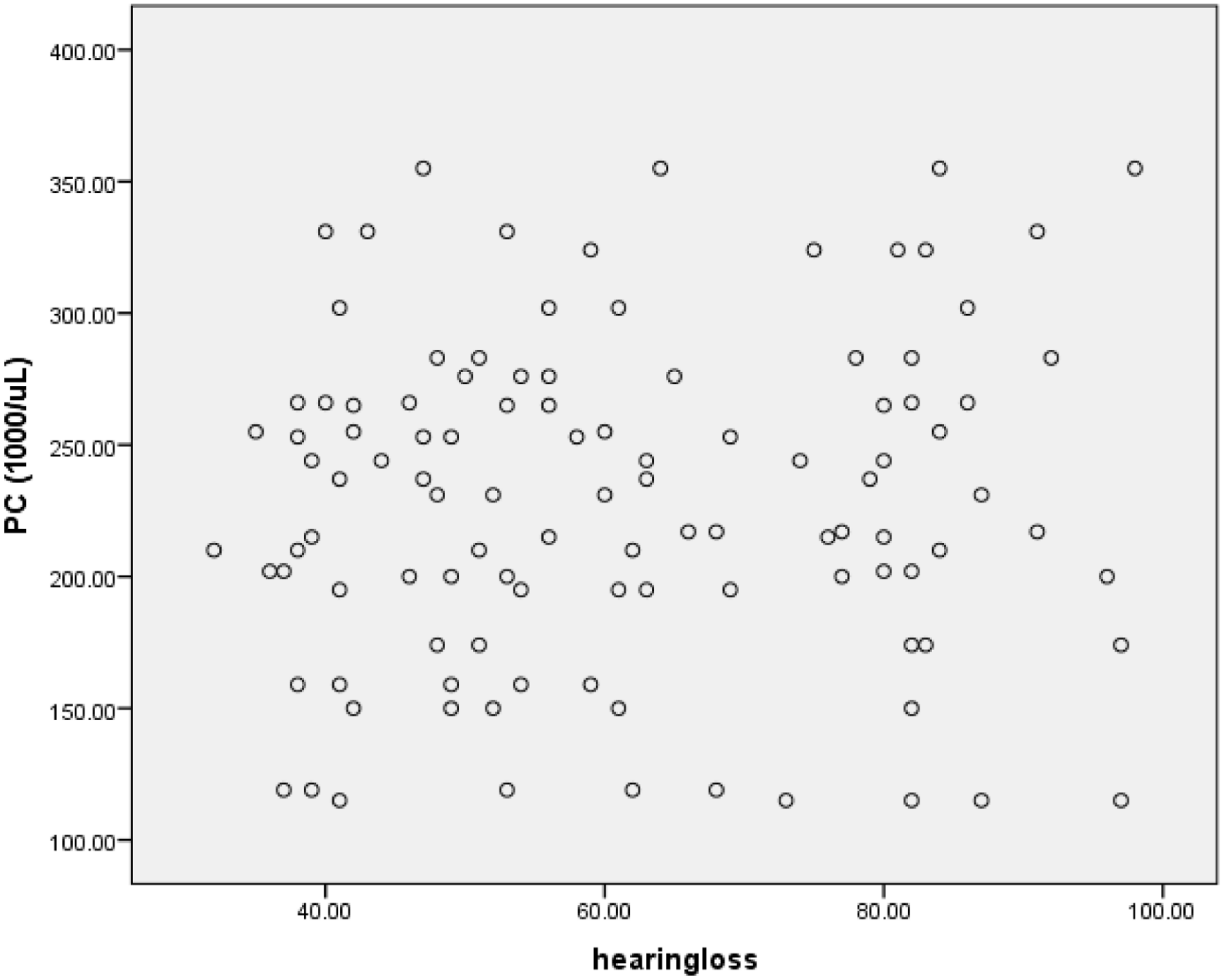
Platelets count versus the severity of hearing loss.

**Fig 5:**
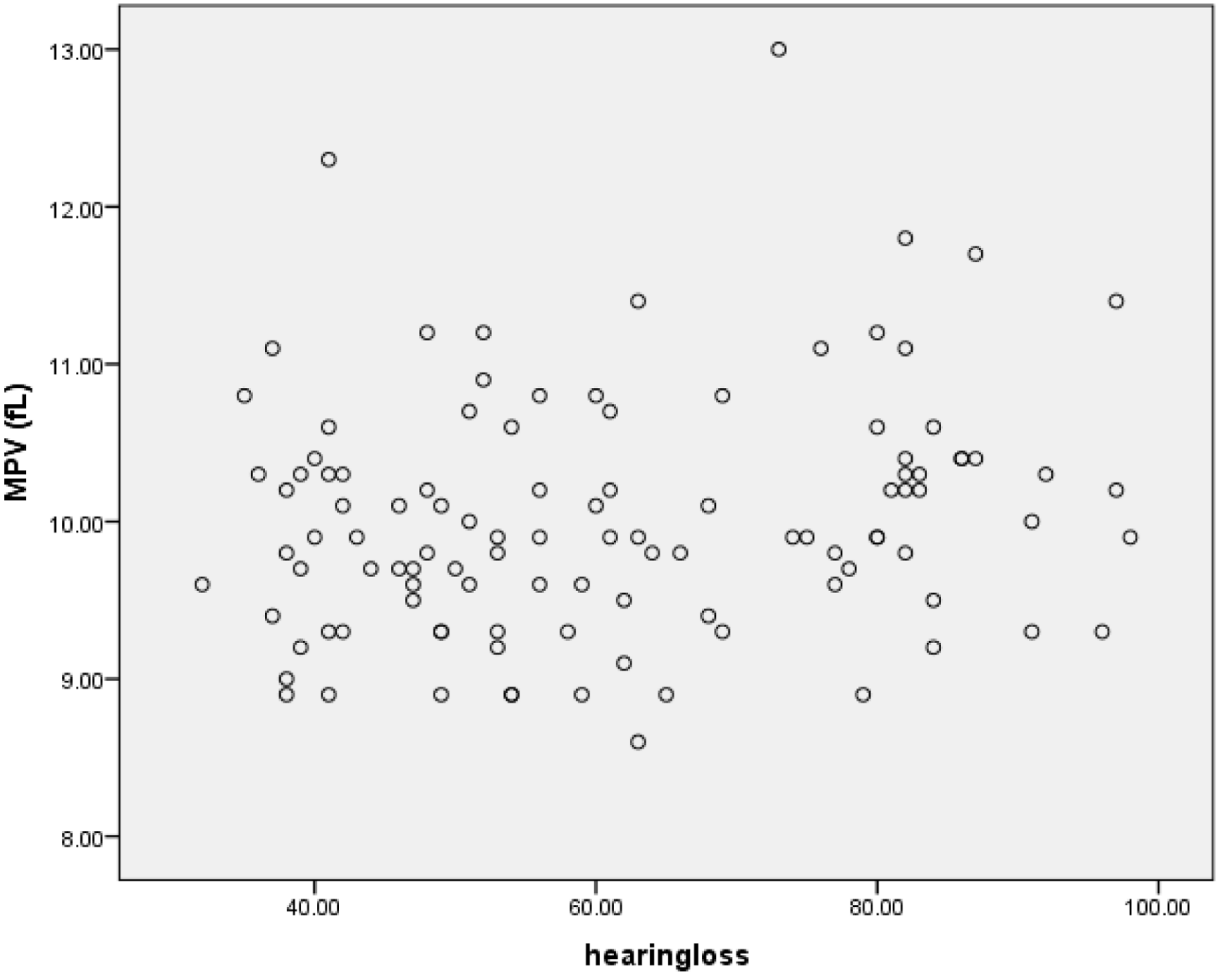
MPV versus the severity of hearing loss.

**Fig 6:**
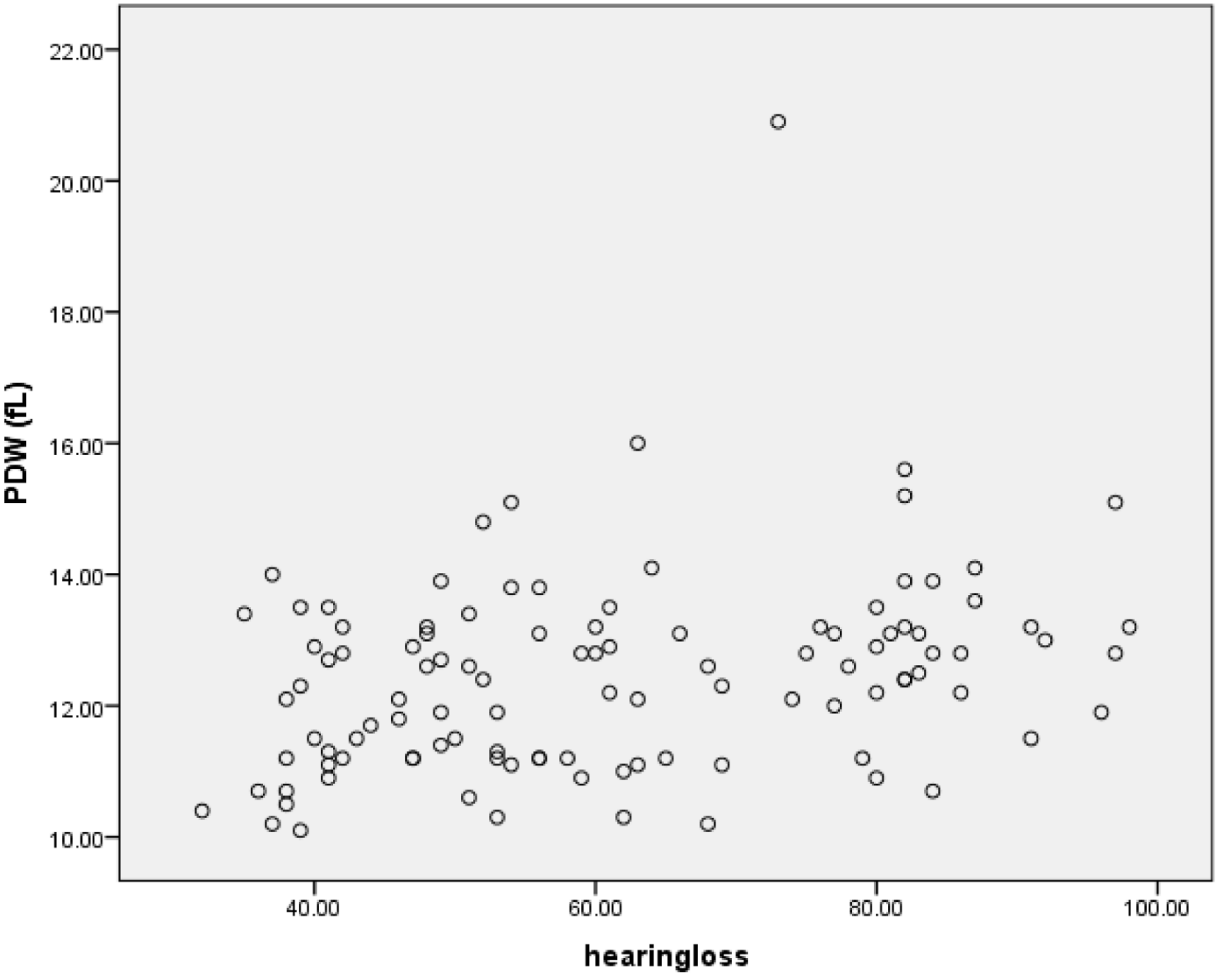
PDW versus the severity of hearing loss.

For more clarity, we draw the scatterplots of platelets parameters versus severity of hearing loss. Figs 4–6 show the PC, MPV and PDW of patients group versus the severity of their hearing loss.

As shown in Table 4, the results showed significant differences between PDW values of patients with different hearing loss levels. For more investigation we used the Pearson correlation coefficients to estimate the correlation between platelet parameters and hearing loss. The results are presented in Table 5:

**Table 5:** Correlations between platelet parameters and hearing loss.

	PC (1000/uL)		MPV (fL)		PDW (fL)	
	r	p-value	r	p-value	r	p-value
**Hearing loss**	0.067	0.488	0.176	0.069	0.299	0.002*

* Correlation is significant at the 0.01 level (2-tailed)

As presented in the above table, PDW showed significant correlation with hearing loss which is seen in scatterplot 3, too.

## Discussion

Sudden sensorineural hearing loss is a common otological disorder with an increasing rate of incidence worldwide, based on current reports. SSNHL requires immediate attention due to its varied etiologic factors and prognostic features [1,34]. Although in most cases, the severity of disease is mild, it can lead to long-term hearing loss, permanent hearing loss, and conditions such as tinnitus [35,36]. The sudden nature of occurrence makes this disorder stressful for the patients, severely affecting their quality of life [18].

Due to the diverse etiology of SSNHL, its treatment has not been satisfactory [37]. In addition, the number of controlled studies on various aspects of this disorder is relatively low due to its very heterogeneous pathology, high rate of spontaneous recovery as well as the delay by many patients in seeking care or referral to medical specialists [8,38].

Previous studies have shown that platelets parameters such as mean platelet volume are associated with ischemic heart events [14], and in some cases, the clinical manifestation of SSNHL is similar to that of vascular diseases—the suddenness of occurrence, the unilateral nature of symptoms and the existence of tinnitus [39]. It has been proposed that the occurrence of SSNHL is related to a disruption of blood supply in the inner ear, because of its sudden and asymptomatic occurrence [24]. In this study, we attempted to investigate the relationship of platelet parameters with the occurrence of SSNHL. Our findings Tables 2 and 3) showed that none of the three examined parameters including PC, MPV and PDW had a significant relationship with the occurrence of SSNHL.

Few studies have examined the relationship of these variables with sudden sensorineural hearing loss. Seo et al. (2014), in a study to identify diagnostic and prognostic biomarkers in patients with SSNHL, compared the biomarker profile of patient and healthy control groups, and found that they differed significantly in their platelet count [27]. However, Blaha et al. (2014) found, in a similar study, that although the values of the mean platelet volume of the two groups did not exhibit any statistically significant relationship with SSNHL, a negative correlation existed between platelet count and SSNHL [29]. Another similar study by Ozcan et al (2014), has not established the existence of a significant correlation between MPV and sudden hearing loss. They found a same result about the correlation between PC and SSNHL [28]. In addition, Ulu et al. (2013) observed that both PDW and MPV exhibited a significant relationship with the occurrence of SSNHL in a case-control study [5]. Segit et al. (2013), in another study, found that the SSHNL patient group exhibited higher MPV values than the control group, but it had no bearing on the severity of the disorder [30]. In contrast, Karli et al. (2013) reported that there was no significant correlation observed between two platelet parameters (PC, MPV) and SSNHL [14]. Also, in this study we compared the platelet parameters according to severity of hearing loss to analyze the relationship between platelet parameters and hearing loss severity in patients group. In this case, our analysis including ANOVA and Pearson correlation coefficients showed a significant correlation between PDW and hearing loss severity. Another similar study by Ozcan et al (2014), has not established the existence of a significant correlation between MPV values and severity of hearing loss (p = 0.701) [28]. Segit et al. (2013), in another study, have reported the same finding about the correlation of MPV and hearing loss severity [30]. In conclusion, the results from our case-control study to determine the relationship between platelet parameters and occurrence and severity of SSHNL, while in agreement with those reported by some previous studies, were in conflict with the results of similar studies by most other groups. Further investigation is required to unequivocally establish the absence of correlation between the two events. It is notable that our study had some strength. The exact selection of patients based on the right inclusion and exclusion criteria and matched controls is the main strength of the study.

## Supporting Information

S1 Table Descriptive and laboratory data of all participants. (DOC)

S2 Table STROBE checklist. (DOC)
