# Correction: Role of Platelet Parameters on Sudden Sensorineural Hearing Loss: A Case-Control Study in Iran

**DOI:** 10.1371/journal.pone.0159695

**Published:** 2016-07-14

**Authors:** Abbas Mirvakili, Mohammad Hossein Dadgarnia, Mohammad Hossein Baradaranfar, Saeid Atighechi, Vahid Zand, Abdollah Ansari

There is an error in reference 28. The correct reference is: Kum RO, Ozcan M, Baklaci D, Yurtsever Kum N, Yilmaz YF, Unal A, Avci Y. Investigation of neutrophil-to-lymphocyte ratio and mean platelet volume in sudden hearing loss. Braz J Otorhinolaryngol. 2015 Nov-Dec; 81(6): 636–41. doi: 10.1016/j.bjorl.2015.08.009. Epub 2015 Sep 7. PubMed PMID: 26480902.
