# Supplementary material for: Role of Platelet Parameters on Sudden Sensorineural Hearing Loss: A Case-Control Study in Iran
Source: PLoS One. 2016 Feb 1;11(2):e0148149. doi: 10.1371/journal.pone.0148149 (PMC4734775; doi:10.1371/journal.pone.0148149)
Supplement: S1 Table — (DOC) [file pone.0148149.s001.doc]

**S1 Table: Descriptive and laboratory data of all participants**

| **No.** | **BMI** | **Age** | **Gender^*^** | **PC (1000/uL)** | **PDW (fL)** | **MPV (fL)** | **Group^**^** | **Hearing loss** | **Hearing loss level^***^** |
| --- | --- | --- | --- | --- | --- | --- | --- | --- | --- |
| **1** | 20.00 | 24.00 | 2.00 | 271.00 | 10.90 | 9.10 | .00 | - | - |
| **2** | 22.00 | 30.00 | 2.00 | 246.00 | 10.70 | 9.20 | .00 | - | - |
| **3** | 21.00 | 35.00 | 2.00 | 304.00 | 11.30 | 9.70 | .00 | - | - |
| **4** | 19.00 | 34.00 | 2.00 | 177.00 | 12.60 | 9.50 | .00 | - | - |
| **5** | 20.00 | 37.00 | 2.00 | 227.00 | 13.90 | 10.10 | .00 | - | - |
| **6** | 24.00 | 41.00 | 2.00 | 271.00 | 14.00 | 11.00 | .00 | - | - |
| **7** | 24.00 | 48.00 | 2.00 | 223.00 | 13.30 | 10.80 | .00 | - | - |
| **8** | 23.00 | 52.00 | 2.00 | 279.00 | 12.60 | 10.20 | .00 | - | - |
| **9** | 25.00 | 55.00 | 2.00 | 197.00 | 13.90 | 10.20 | .00 | - | - |
| **10** | 24.00 | 57.00 | 2.00 | 244.00 | 11.80 | 9.80 | .00 | - | - |
| **11** | 23.00 | 48.00 | 2.00 | 202.00 | 11.70 | 9.30 | .00 | - | - |
| **12** | 20.00 | 22.00 | 1.00 | 216.00 | 11.10 | 8.90 | .00 | - | - |
| **13** | 22.00 | 24.00 | 1.00 | 206.00 | 11.30 | 9.10 | .00 | - | - |
| **14** | 21.00 | 27.00 | 1.00 | 228.00 | 9.70 | 8.40 | .00 | - | - |
| **15** | 24.00 | 41.00 | 1.00 | 174.00 | 11.90 | 9.70 | .00 | - | - |
| **16** | 25.00 | 41.00 | 1.00 | 210.00 | 12.90 | 10.30 | .00 | - | - |
| **17** | 23.00 | 44.00 | 1.00 | 195.00 | 14.00 | 9.90 | .00 | - | - |
| **18** | 24.00 | 45.00 | 1.00 | 269.00 | 10.30 | 8.30 | .00 | - | - |
| **19** | 23.00 | 52.00 | 1.00 | 212.00 | 10.00 | 8.30 | .00 | - | - |
| **20** | 24.00 | 52.00 | 1.00 | 174.00 | 13.20 | 10.40 | .00 | - | - |
| **21** | 25.00 | 55.00 | 1.00 | 168.00 | 13.50 | 10.70 | .00 | - | - |
| **22** | 23.00 | 58.00 | 1.00 | 222.00 | 9.80 | 8.50 | .00 | - | - |
| **23** | 24.00 | 42.00 | 1.00 | 194.00 | 12.00 | 9.70 | .00 | - | - |
| **24** | 25.00 | 68.00 | 1.00 | 194.00 | 11.00 | 9.70 | .00 | - | - |
| **25** | 22.00 | 26.00 | 2.00 | 271.00 | 11.30 | 9.40 | .00 | - | - |
| **26** | 24.00 | 37.00 | 2.00 | 246.00 | 11.00 | 9.60 | .00 | - | - |
| **27** | 23.00 | 33.00 | 2.00 | 304.00 | 11.40 | 9.70 | .00 | - | - |
| **28** | 23.00 | 36.00 | 2.00 | 177.00 | 12.70 | 10.60 | .00 | - | - |
| **29** | 23.00 | 35.00 | 2.00 | 227.00 | 13.90 | 10.20 | .00 | - | - |
| **30** | 21.00 | 42.00 | 2.00 | 271.00 | 14.10 | 11.20 | .00 | - | - |
| **31** | 24.00 | 48.00 | 2.00 | 223.00 | 13.20 | 10.80 | .00 | - | - |
| **32** | 24.00 | 51.00 | 2.00 | 279.00 | 12.90 | 9.50 | .00 | - | - |
| **33** | 24.00 | 54.00 | 2.00 | 197.00 | 13.60 | 10.40 | .00 | - | - |
| **34** | 25.00 | 59.00 | 2.00 | 244.00 | 12.00 | 10.30 | .00 | - | - |
| **35** | 21.00 | 49.00 | 2.00 | 202.00 | 11.90 | 9.60 | .00 | - | - |
| **36** | 22.00 | 26.00 | 1.00 | 216.00 | 11.60 | 9.80 | .00 | - | - |
| **37** | 22.00 | 23.00 | 1.00 | 206.00 | 11.40 | 9.40 | .00 | - | - |
| **38** | 21.00 | 25.00 | 1.00 | 228.00 | 10.50 | 9.20 | .00 | - | - |
| **39** | 23.00 | 43.00 | 1.00 | 174.00 | 11.90 | 9.80 | .00 | - | - |
| **40** | 23.00 | 42.00 | 1.00 | 210.00 | 12.90 | 10.60 | .00 | - | - |
| **41** | 22.00 | 39.00 | 1.00 | 195.00 | 13.90 | 9.60 | .00 | - | - |
| **42** | 25.00 | 50.00 | 1.00 | 269.00 | 10.80 | 9.10 | .00 | - | - |
| **43** | 24.00 | 52.00 | 1.00 | 212.00 | 10.40 | 9.10 | .00 | - | - |
| **44** | 26.00 | 52.00 | 1.00 | 174.00 | 13.20 | 11.40 | .00 | - | - |
| **45** | 25.00 | 55.00 | 1.00 | 168.00 | 13.40 | 10.70 | .00 | - | - |
| **46** | 26.00 | 25.00 | 1.00 | 222.00 | 9.80 | 9.30 | .00 | - | - |
| **47** | 22.00 | 42.00 | 1.00 | 194.00 | 12.40 | 9.70 | .00 | - | - |
| **48** | 25.00 | 68.00 | 1.00 | 194.00 | 11.20 | 9.90 | .00 | - | - |
| **49** | 24.00 | 58.00 | 2.00 | 271.00 | 11.10 | 9.40 | .00 | - | - |
| **50** | 20.00 | 31.00 | 2.00 | 246.00 | 10.80 | 9.30 | .00 | - | - |
| **51** | 22.00 | 35.00 | 2.00 | 304.00 | 11.40 | 9.70 | .00 | - | - |
| **52** | 21.00 | 34.00 | 2.00 | 177.00 | 12.30 | 9.80 | .00 | - | - |
| **53** | 20.00 | 39.00 | 2.00 | 227.00 | 13.70 | 10.20 | .00 | - | - |
| **54** | 24.00 | 42.00 | 2.00 | 271.00 | 13.90 | 11.20 | .00 | - | - |
| **55** | 22.00 | 49.00 | 2.00 | 223.00 | 13.10 | 11.40 | .00 | - | - |
| **56** | 23.00 | 50.00 | 2.00 | 279.00 | 12.70 | 9.70 | .00 | - | - |
| **57** | 24.00 | 55.00 | 2.00 | 197.00 | 13.80 | 10.40 | .00 | - | - |
| **58** | 25.00 | 55.00 | 2.00 | 244.00 | 11.80 | 9.90 | .00 | - | - |
| **59** | 24.00 | 48.00 | 2.00 | 202.00 | 11.90 | 9.60 | .00 | - | - |
| **60** | 22.00 | 41.00 | 1.00 | 216.00 | 11.30 | 9.30 | .00 | - | - |
| **61** | 24.00 | 24.00 | 1.00 | 206.00 | 11.40 | 9.10 | .00 | - | - |
| **62** | 21.00 | 28.00 | 1.00 | 228.00 | 9.90 | 9.20 | .00 | - | - |
| **63** | 23.00 | 41.00 | 1.00 | 174.00 | 11.90 | 9.70 | .00 | - | - |
| **64** | 23.00 | 27.00 | 1.00 | 210.00 | 12.70 | 10.50 | .00 | - | - |
| **65** | 24.00 | 44.00 | 1.00 | 195.00 | 14.10 | 9.90 | .00 | - | - |
| **66** | 22.00 | 48.00 | 1.00 | 269.00 | 11.10 | 8.70 | .00 | - | - |
| **67** | 26.00 | 51.00 | 1.00 | 212.00 | 10.40 | 9.10 | .00 | - | - |
| **68** | 25.00 | 52.00 | 1.00 | 174.00 | 13.20 | 10.50 | .00 | - | - |
| **69** | 25.00 | 53.00 | 1.00 | 168.00 | 13.30 | 10.80 | .00 | - | - |
| **70** | 24.00 | 58.00 | 1.00 | 222.00 | 9.90 | 9.20 | .00 | - | - |
| **71** | 23.00 | 40.00 | 1.00 | 194.00 | 12.10 | 10.10 | .00 | - | - |
| **72** | 25.00 | 68.00 | 1.00 | 194.00 | 11.20 | 9.60 | .00 | - | - |
| **73** | 24.00 | 23.00 | 2.00 | 271.00 | 10.90 | 9.70 | .00 | - | - |
| **74** | 22.00 | 33.00 | 2.00 | 246.00 | 11.30 | 9.80 | .00 | - | - |
| **75** | 21.00 | 35.00 | 2.00 | 304.00 | 11.60 | 9.90 | .00 | - | - |
| **76** | 22.00 | 35.00 | 2.00 | 177.00 | 12.70 | 9.50 | .00 | - | - |
| **77** | 20.00 | 38.00 | 2.00 | 227.00 | 13.60 | 10.10 | .00 | - | - |
| **78** | 22.00 | 44.00 | 2.00 | 271.00 | 14.00 | 11.00 | .00 | - | - |
| **79** | 23.00 | 46.00 | 2.00 | 223.00 | 13.20 | 10.80 | .00 | - | - |
| **80** | 24.00 | 51.00 | 2.00 | 279.00 | 12.80 | 10.50 | .00 | - | - |
| **81** | 23.00 | 54.00 | 2.00 | 197.00 | 13.80 | 9.90 | .00 | - | - |
| **82** | 26.00 | 58.00 | 2.00 | 244.00 | 12.20 | 10.20 | .00 | - | - |
| **83** | 21.00 | 48.00 | 2.00 | 202.00 | 11.80 | 9.80 | .00 | - | - |
| **84** | 22.00 | 24.00 | 1.00 | 216.00 | 11.40 | 8.90 | .00 | - | - |
| **85** | 24.00 | 28.00 | 1.00 | 206.00 | 11.30 | 9.40 | .00 | - | - |
| **86** | 24.00 | 29.00 | 1.00 | 228.00 | 10.60 | 9.40 | .00 | - | - |
| **87** | 23.00 | 39.00 | 1.00 | 174.00 | 11.90 | 9.70 | .00 | - | - |
| **88** | 24.00 | 46.00 | 1.00 | 210.00 | 13.20 | 10.30 | .00 | - | - |
| **89** | 22.00 | 44.00 | 1.00 | 195.00 | 14.00 | 10.10 | .00 | - | - |
| **90** | 23.00 | 42.00 | 1.00 | 269.00 | 10.60 | 8.60 | .00 | - | - |
| **91** | 25.00 | 53.00 | 1.00 | 212.00 | 10.00 | 9.50 | .00 | - | - |
| **92** | 24.00 | 52.00 | 1.00 | 174.00 | 13.20 | 10.20 | .00 | - | - |
| **93** | 26.00 | 57.00 | 1.00 | 168.00 | 13.40 | 10.90 | .00 | - | - |
| **94** | 24.00 | 58.00 | 1.00 | 222.00 | 9.90 | 9.70 | .00 | - | - |
| **95** | 24.00 | 40.00 | 1.00 | 194.00 | 12.60 | 9.80 | .00 | - | - |
| **96** | 26.00 | 69.00 | 1.00 | 194.00 | 11.20 | 9.90 | .00 | - | - |
| **97** | 21.00 | 29.00 | 1.00 | 271.00 | 10.90 | 9.30 | .00 | - | - |
| **98** | 22.00 | 32.00 | 1.00 | 246.00 | 11.00 | 9.70 | .00 | - | - |
| **99** | 25.00 | 36.00 | 1.00 | 304.00 | 11.40 | 9.90 | .00 | - | - |
| **100** | 23.00 | 32.00 | 1.00 | 177.00 | 12.60 | 10.00 | .00 | - | - |
| **101** | 25.00 | 38.00 | 1.00 | 227.00 | 13.70 | 10.60 | .00 | - | - |
| **102** | 26.00 | 41.00 | 1.00 | 271.00 | 14.00 | 11.20 | .00 | - | - |
| **103** | 24.00 | 53.00 | 1.00 | 223.00 | 13.10 | 10.80 | .00 | - | - |
| **104** | 25.00 | 51.00 | 1.00 | 279.00 | 12.60 | 10.40 | .00 | - | - |
| **105** | 24.00 | 58.00 | 1.00 | 197.00 | 13.80 | 10.20 | .00 | - | - |
| **106** | 24.00 | 57.00 | 2.00 | 244.00 | 12.40 | 9.80 | .00 | - | - |
| **107** | 25.00 | 46.00 | 2.00 | 202.00 | 12.30 | 9.80 | .00 | - | - |
| **108** | 21.00 | 27.00 | 2.00 | 216.00 | 11.80 | 8.90 | .00 | - | - |
| **109** | 24.00 | 33.00 | 1.00 | 119.00 | 10.10 | 9.20 | 1.00 | 39.00 | 1.00 |
| **110** | 25.00 | 69.00 | 2.00 | 202.00 | 10.70 | 10.30 | 1.00 | 36.00 | 1.00 |
| **111** | 25.00 | 49.00 | 1.00 | 266.00 | 12.10 | 10.20 | 1.00 | 38.00 | 1.00 |
| **112** | 24.00 | 57.00 | 1.00 | 244.00 | 12.30 | 9.70 | 1.00 | 39.00 | 1.00 |
| **113** | 25.00 | 69.00 | 2.00 | 159.00 | 10.70 | 8.90 | 1.00 | 38.00 | 1.00 |
| **114** | 24.00 | 25.00 | 2.00 | 331.00 | 11.50 | 9.90 | 1.00 | 40.00 | 1.00 |
| **115** | 22.00 | 28.00 | 1.00 | 210.00 | 10.50 | 9.00 | 1.00 | 38.00 | 1.00 |
| **116** | 24.00 | 34.00 | 1.00 | 119.00 | 10.20 | 9.40 | 1.00 | 37.00 | 1.00 |
| **117** | 24.00 | 24.00 | 1.00 | 210.00 | 10.40 | 9.60 | 1.00 | 32.00 | 1.00 |
| **118** | 23.00 | 55.00 | 1.00 | 215.00 | 13.50 | 10.30 | 1.00 | 39.00 | 1.00 |
| **119** | 22.00 | 29.00 | 1.00 | 253.00 | 11.20 | 9.80 | 1.00 | 38.00 | 1.00 |
| **120** | 23.00 | 46.00 | 2.00 | 255.00 | 13.40 | 10.80 | 1.00 | 35.00 | 1.00 |
| **121** | 24.00 | 70.00 | 1.00 | 202.00 | 14.00 | 11.10 | 1.00 | 37.00 | 1.00 |
| **122** | 25.00 | 33.00 | 1.00 | 217.00 | 12.60 | 10.10 | 1.00 | 68.00 | 2.00 |
| **123** | 23.00 | 26.00 | 1.00 | 253.00 | 11.20 | 9.60 | 1.00 | 47.00 | 2.00 |
| **124** | 23.00 | 60.00 | 2.00 | 276.00 | 11.10 | 8.90 | 1.00 | 54.00 | 2.00 |
| **125** | 25.00 | 63.00 | 1.00 | 115.00 | 11.30 | 12.30 | 1.00 | 41.00 | 2.00 |
| **126** | 23.00 | 37.00 | 2.00 | 265.00 | 11.20 | 9.30 | 1.00 | 53.00 | 2.00 |
| **127** | 20.00 | 23.00 | 1.00 | 174.00 | 13.40 | 10.70 | 1.00 | 51.00 | 2.00 |
| **128** | 24.00 | 49.00 | 2.00 | 266.00 | 12.10 | 10.10 | 1.00 | 46.00 | 2.00 |
| **129** | 23.00 | 52.00 | 1.00 | 266.00 | 12.90 | 10.40 | 1.00 | 40.00 | 2.00 |
| **130** | 26.00 | 43.00 | 1.00 | 283.00 | 12.60 | 10.00 | 1.00 | 51.00 | 2.00 |
| **131** | 24.00 | 55.00 | 1.00 | 150.00 | 12.70 | 10.10 | 1.00 | 49.00 | 2.00 |
| **132** | 25.00 | 32.00 | 1.00 | 200.00 | 11.80 | 9.70 | 1.00 | 46.00 | 2.00 |
| **133** | 25.00 | 60.00 | 1.00 | 276.00 | 11.50 | 9.70 | 1.00 | 50.00 | 2.00 |
| **134** | 25.00 | 54.00 | 2.00 | 355.00 | 12.90 | 9.70 | 1.00 | 47.00 | 2.00 |
| **135** | 26.00 | 57.00 | 1.00 | 244.00 | 11.70 | 9.70 | 1.00 | 44.00 | 2.00 |
| **136** | 21.00 | 37.00 | 1.00 | 200.00 | 11.90 | 9.30 | 1.00 | 49.00 | 2.00 |
| **137** | 20.00 | 36.00 | 2.00 | 265.00 | 11.20 | 9.30 | 1.00 | 42.00 | 2.00 |
| **137** | 22.00 | 29.00 | 1.00 | 253.00 | 11.40 | 9.30 | 1.00 | 49.00 | 2.00 |
| **139** | 26.00 | 46.00 | 1.00 | 283.00 | 12.60 | 9.80 | 1.00 | 48.00 | 2.00 |
| **140** | 23.00 | 70.00 | 1.00 | 237.00 | 10.90 | 8.90 | 1.00 | 41.00 | 2.00 |
| **141** | 24.00 | 68.00 | 1.00 | 159.00 | 11.10 | 9.30 | 1.00 | 41.00 | 2.00 |
| **142** | 22.00 | 68.00 | 2.00 | 237.00 | 11.20 | 9.50 | 1.00 | 47.00 | 2.00 |
| **143** | 23.00 | 30.00 | 2.00 | 331.00 | 11.30 | 9.90 | 1.00 | 53.00 | 2.00 |
| **144** | 23.00 | 31.00 | 1.00 | 210.00 | 10.60 | 9.60 | 1.00 | 51.00 | 2.00 |
| **145** | 22.00 | 23.00 | 1.00 | 331.00 | 11.50 | 9.90 | 1.00 | 43.00 | 2.00 |
| **146** | 22.00 | 36.00 | 1.00 | 200.00 | 11.90 | 9.80 | 1.00 | 53.00 | 2.00 |
| **147** | 25.00 | 41.00 | 1.00 | 195.00 | 13.80 | 10.60 | 1.00 | 54.00 | 2.00 |
| **148** | 24.00 | 32.00 | 2.00 | 119.00 | 10.30 | 9.20 | 1.00 | 53.00 | 2.00 |
| **149** | 21.00 | 31.00 | 1.00 | 302.00 | 12.70 | 10.30 | 1.00 | 41.00 | 2.00 |
| **150** | 23.00 | 50.00 | 1.00 | 150.00 | 12.80 | 10.10 | 1.00 | 42.00 | 2.00 |
| **151** | 24.00 | 46.00 | 2.00 | 255.00 | 13.20 | 10.30 | 1.00 | 42.00 | 2.00 |
| **152** | 21.00 | 49.00 | 2.00 | 231.00 | 14.80 | 11.20 | 1.00 | 52.00 | 2.00 |
| **153** | 25.00 | 68.00 | 1.00 | 174.00 | 13.20 | 10.20 | 1.00 | 48.00 | 2.00 |
| **154** | 24.00 | 49.00 | 2.00 | 231.00 | 13.10 | 11.20 | 1.00 | 48.00 | 2.00 |
| **155** | 24.00 | 35.00 | 2.00 | 324.00 | 12.80 | 9.60 | 1.00 | 59.00 | 2.00 |
| **156** | 27.00 | 67.00 | 2.00 | 159.00 | 13.90 | 8.90 | 1.00 | 49.00 | 2.00 |
| **157** | 23.00 | 69.00 | 1.00 | 159.00 | 15.10 | 8.90 | 1.00 | 54.00 | 2.00 |
| **158** | 22.00 | 54.00 | 2.00 | 150.00 | 12.40 | 10.90 | 1.00 | 52.00 | 2.00 |
| **159** | 24.00 | 55.00 | 2.00 | 150.00 | 12.20 | 10.20 | 1.00 | 61.00 | 3.00 |
| **160** | 25.00 | 38.00 | 2.00 | 200.00 | 12.00 | 9.60 | 1.00 | 77.00 | 3.00 |
| **161** | 24.00 | 50.00 | 1.00 | 215.00 | 12.90 | 10.60 | 1.00 | 80.00 | 3.00 |
| **162** | 24.00 | 35.00 | 2.00 | 217.00 | 13.10 | 9.80 | 1.00 | 77.00 | 3.00 |
| **163** | 23.00 | 55.00 | 2.00 | 215.00 | 13.80 | 10.80 | 1.00 | 56.00 | 3.00 |
| **164** | 25.00 | 56.00 | 2.00 | 244.00 | 12.10 | 9.90 | 1.00 | 74.00 | 3.00 |
| **165** | 22.00 | 28.00 | 1.00 | 253.00 | 11.10 | 9.30 | 1.00 | 69.00 | 3.00 |
| **166** | 24.00 | 58.00 | 2.00 | 276.00 | 11.20 | 9.60 | 1.00 | 56.00 | 3.00 |
| **167** | 24.00 | 69.00 | 1.00 | 159.00 | 10.90 | 8.90 | 1.00 | 59.00 | 3.00 |
| **168** | 20.00 | 30.00 | 1.00 | 210.00 | 11.00 | 9.10 | 1.00 | 62.00 | 3.00 |
| **169** | 26.00 | 63.00 | 1.00 | 276.00 | 11.20 | 8.90 | 1.00 | 65.00 | 3.00 |
| **170** | 24.00 | 27.00 | 1.00 | 253.00 | 11.20 | 9.30 | 1.00 | 58.00 | 3.00 |
| **171** | 25.00 | 47.00 | 2.00 | 283.00 | 12.60 | 9.70 | 1.00 | 78.00 | 3.00 |
| **172** | 22.00 | 33.00 | 2.00 | 217.00 | 13.10 | 9.80 | 1.00 | 66.00 | 3.00 |
| **173** | 23.00 | 53.00 | 2.00 | 215.00 | 13.20 | 11.10 | 1.00 | 76.00 | 3.00 |
| **174** | 25.00 | 34.00 | 2.00 | 265.00 | 11.20 | 9.90 | 1.00 | 56.00 | 3.00 |
| **175** | 23.00 | 39.00 | 2.00 | 324.00 | 12.80 | 9.90 | 1.00 | 75.00 | 3.00 |
| **176** | 22.00 | 55.00 | 1.00 | 244.00 | 12.10 | 9.90 | 1.00 | 63.00 | 3.00 |
| **177** | 23.00 | 64.00 | 1.00 | 115.00 | 20.90 | 13.00 | 1.00 | 73.00 | 3.00 |
| **178** | 21.00 | 33.00 | 2.00 | 302.00 | 13.10 | 10.20 | 1.00 | 56.00 | 3.00 |
| **179** | 22.00 | 36.00 | 1.00 | 119.00 | 10.20 | 9.40 | 1.00 | 68.00 | 3.00 |
| **180** | 24.00 | 41.00 | 1.00 | 195.00 | 13.50 | 10.60 | 1.00 | 41.00 | 3.00 |
| **181** | 20.00 | 21.00 | 1.00 | 237.00 | 11.10 | 8.60 | 1.00 | 63.00 | 3.00 |
| **182** | 22.00 | 30.00 | 1.00 | 119.00 | 10.30 | 9.50 | 1.00 | 62.00 | 3.00 |
| **183** | 26.00 | 52.00 | 1.00 | 355.00 | 14.10 | 9.80 | 1.00 | 64.00 | 3.00 |
| **184** | 23.00 | 41.00 | 2.00 | 195.00 | 13.50 | 10.70 | 1.00 | 61.00 | 3.00 |
| **185** | 24.00 | 68.00 | 2.00 | 237.00 | 11.20 | 8.90 | 1.00 | 79.00 | 3.00 |
| **186** | 24.00 | 42.00 | 1.00 | 195.00 | 12.30 | 10.80 | 1.00 | 69.00 | 3.00 |
| **187** | 24.00 | 51.00 | 1.00 | 231.00 | 12.80 | 10.80 | 1.00 | 60.00 | 3.00 |
| **188** | 21.00 | 30.00 | 2.00 | 302.00 | 12.90 | 9.90 | 1.00 | 61.00 | 3.00 |
| **189** | 24.00 | 41.00 | 1.00 | 195.00 | 16.00 | 11.40 | 1.00 | 63.00 | 3.00 |
| **190** | 21.00 | 49.00 | 2.00 | 255.00 | 13.20 | 10.10 | 1.00 | 60.00 | 3.00 |
| **191** | 25.00 | 66.00 | 2.00 | 115.00 | 13.60 | 11.70 | 1.00 | 87.00 | 3.00 |
| **192** | 25.00 | 32.00 | 2.00 | 302.00 | 12.80 | 10.40 | 1.00 | 86.00 | 3.00 |
| **193** | 22.00 | 26.00 | 1.00 | 174.00 | 12.50 | 10.30 | 1.00 | 83.00 | 3.00 |
| **194** | 28.00 | 72.00 | 2.00 | 202.00 | 13.50 | 11.20 | 1.00 | 80.00 | 3.00 |
| **195** | 25.00 | 57.00 | 2.00 | 355.00 | 12.80 | 9.50 | 1.00 | 84.00 | 3.00 |
| **196** | 25.00 | 43.00 | 1.00 | 283.00 | 12.40 | 9.80 | 1.00 | 82.00 | 3.00 |
| **197** | 24.00 | 45.00 | 2.00 | 255.00 | 13.90 | 10.60 | 1.00 | 84.00 | 3.00 |
| **198** | 24.00 | 52.00 | 2.00 | 244.00 | 12.20 | 9.90 | 1.00 | 80.00 | 3.00 |
| **199** | 18.00 | 27.00 | 1.00 | 210.00 | 10.70 | 9.20 | 1.00 | 84.00 | 3.00 |
| **200** | 25.00 | 70.00 | 1.00 | 202.00 | 15.20 | 11.10 | 1.00 | 82.00 | 3.00 |
| **201** | 24.00 | 36.00 | 2.00 | 324.00 | 13.10 | 10.20 | 1.00 | 81.00 | 3.00 |
| **202** | 22.00 | 44.00 | 2.00 | 231.00 | 14.10 | 10.40 | 1.00 | 87.00 | 3.00 |
| **203** | 22.00 | 50.00 | 1.00 | 266.00 | 12.40 | 10.20 | 1.00 | 82.00 | 3.00 |
| **204** | 23.00 | 22.00 | 1.00 | 174.00 | 13.90 | 10.40 | 1.00 | 82.00 | 3.00 |
| **205** | 25.00 | 55.00 | 1.00 | 150.00 | 13.20 | 10.30 | 1.00 | 82.00 | 3.00 |
| **206** | 22.00 | 35.00 | 1.00 | 265.00 | 10.90 | 9.90 | 1.00 | 80.00 | 3.00 |
| **207** | 21.00 | 33.00 | 2.00 | 324.00 | 13.10 | 10.20 | 1.00 | 83.00 | 3.00 |
| **208** | 25.00 | 51.00 | 1.00 | 266.00 | 12.20 | 10.40 | 1.00 | 86.00 | 3.00 |
| **209** | 24.00 | 60.00 | 2.00 | 115.00 | 15.60 | 11.80 | 1.00 | 82.00 | 3.00 |
| **210** | 22.00 | 23.00 | 2.00 | 331.00 | 11.50 | 9.30 | 1.00 | 91.00 | 4.00 |
| **211** | 26.00 | 55.00 | 2.00 | 355.00 | 13.20 | 9.90 | 1.00 | 98.00 | 4.00 |
| **212** | 23.00 | 44.00 | 1.00 | 283.00 | 13.00 | 10.30 | 1.00 | 92.00 | 4.00 |
| **213** | 24.00 | 39.00 | 1.00 | 200.00 | 11.90 | 9.30 | 1.00 | 96.00 | 4.00 |
| **214** | 24.00 | 22.00 | 1.00 | 174.00 | 12.80 | 10.20 | 1.00 | 97.00 | 4.00 |
| **215** | 22.00 | 33.00 | 2.00 | 217.00 | 13.20 | 10.00 | 1.00 | 91.00 | 4.00 |
| **216** | 26.00 | 64.00 | 1.00 | 115.00 | 15.10 | 11.40 | 1.00 | 97.00 | 4.00 |

**^*^Gender: 1=Male, 2=Female**

**^**^Group: 0=Control, 2=Patient**

**^***^Hearing loss level: 1=Mild, 2=Moderate, 3=Sever, 4=Profound**
